# Schistosomiasis Seroprevalence among Children Aged 0–14 Years in Nigeria, 2018

**DOI:** 10.4269/ajtmh.23-0219

**Published:** 2023-11-27

**Authors:** Anne Straily, Israel Tamunonengiyeofori, Ryan E. Wiegand, Nnaemeka C. Iriemenam, McPaul I. Okoye, Ayuba B. Dawurung, Nkechi Blessing Ugboaja, Martha Tongha, Nishanth Parameswaran, Stacie M. Greby, Matthias Alagi, Nseobong M. Akpan, William E. Nwachukwu, Nwando Mba, Diana L. Martin, W. Evan Secor, Mahesh Swaminathan, Ifedayo Adetifa, Chikwe Ihekweazu

**Affiliations:** ^1^ Division of Parasitic Diseases and Malaria, Centers for Disease Control and Prevention, Atlanta, Georgia;; ^2^ Nigeria Centre for Disease Control and Prevention, Abuja, Nigeria;; ^3^ Division of Global HIV and TB, Centers for Disease Control and Prevention, Abuja, Nigeria;; ^4^ Institute of Human Virology, Abuja, Nigeria;; ^5^ Neglected Tropical Diseases, Federal Ministry of Health, Abuja, Nigeria

## Abstract

The first nationally representative, population-based study of schistosomiasis seroprevalence in Nigeria was conducted using blood samples and risk-factor data collected during the 2018 Nigeria HIV/AIDS Indicator and Impact Survey (NAIIS). Schistosomiasis seroprevalence was estimated by analyzing samples for reactivity to schistosome soluble egg antigen (SEA) in a multiplex bead assay; NAIIS survey data were assessed to identify potential risk factors for seropositivity. The SEA antibody data were available for 31,459 children aged 0 to 14 years. Overall seroprevalence was 17.2% (95% CI: 16.3–18.1%). Seropositive children were identified in every age group, including children < 5 years, and seroprevalence increased with increasing age (*P <* 0.0001). Several factors were associated with increased odds of seropositivity, including being a boy (odds ratio [OR] = 1.34, 95% CI: 1.24–1.45), living in a rural area (OR = 2.2, 95% CI: 1.9–2.5), and animal ownership (OR = 1.67, 95% CI: 1.52–1.85). Access to improved sanitation and drinking water sources were associated with decreased odds of seropositivity (OR = 0.52, 95% CI: 0.47–0.58 and OR = 0.53, 95% CI: 0.47–0.60, respectively) regardless of whether the child lived in a rural (sanitation: adjusted odds ratio [aOR] = 0.7, 95% CI: 0.6–0.8; drinking water: aOR = 0.7, 95% CI: 0.6–0.8) or urban area (sanitation: aOR = 0.6, 95% CI: 0.5–0.7; drinking water: aOR = 0.5, 95% CI: 0.4–0.6), highlighting the importance of these factors for schistosomiasis prevention and control. These results identified additional risk populations (children < 5 years) and a new risk factor (animal ownership) and could be used to monitor the impact of control programs.

## INTRODUCTION

Schistosomiasis is a neglected tropical disease caused by infection with *Schistosoma* sp. trematodes. Depending on the infecting species, chronic schistosomiasis can cause intestinal or urogenital disease. Nigeria is estimated to have the highest prevalence of schistosomiasis among all countries in sub-Saharan Africa (which is home to an estimated 93% of schistosomiasis cases globally).^1^^,^^2^ *Schistosoma haematobium*, the cause of urogenital schistosomiasis, is the predominant species in Nigeria, accounting for almost 80% of cases.^3^ Urogenital schistosomiasis has been associated with female genital schistosomiasis, an increased risk of HIV transmission, squamous cell carcinomas in the bladder, and cervical cancers.^4^ The remaining schistosome infections are intestinal cases primarily attributable to *Schistosoma mansoni* and a small number of *Schistosoma intercalatum* (< 5%) infections.^3^

Surveys to determine schistosomiasis prevalence and intensity by traditional stool or urine-based methods such as Kato Katz^5^ and urine filtration^5^ are costly^6^^,^^7^ and time-consuming and are rarely undertaken on a national level to produce population-representative estimates. Often only one stool or urine sample is collected and examined, which reduces sensitivity in both test types and could bias resulting prevalence estimates. Disease burden estimates produced for particular sites based on directed research efforts or periodic surveys are lacking for areas where these research efforts are not conducted and may collect data using different study designs. As a result, research study data are sometimes less useful for producing a reliable national estimate.^8^ In 2018, Nigeria conducted the Nigeria HIV/AIDS Indicator and Impact Survey (NAIIS), a nationally representative household HIV survey that included collection and storage of blood specimens. Representative surveys like the NAIIS provide an opportunity to gain information on seroprevalence for other diseases of interest. Serological data can provide a snapshot of the epidemiological context of the disease at the time of the survey or could be used longitudinally to measure the changing intensity of transmission.^9^ Multiplex serologic assays, which can measure antibody levels for multiple pathogens simultaneously in a single sample, provide a more efficient and cost-effective means of obtaining national, population-based estimates of disease burden.^10^ Multiplex assays offer some additional advantages for seroprevalence studies: The assays themselves can be modified as needed for individual countries’ program objectives and local disease profiles, they require only a small volume of blood (1 µL or less of serum), and they can also be performed on dried blood spots (DBSs).^10^ These low-volume specimen requirements are especially advantageous for studies that include children.^10^

Evaluations of schistosomiasis serology in sub-Saharan Africa have identified a strong correlation between *S. mansoni* soluble egg antigen (SEA) seroprevalence and infection prevalence identified by microscopy.^9^^,^^11^ We analyzed DBS specimens collected during the 2018 NAIIS for serologic reactivity to SEA. The objectives of these analyses were 2-fold: first, to produce national-level estimates for the prevalence of schistosomiasis by measuring antibody responses to SEA among children < 15 years of age in Nigeria and second, to identify risk factors related to schistosomiasis seropositivity using the survey data.

## MATERIALS AND METHODS

### Survey and specimens.

During the 2018 NAIIS, blood specimens were collected and informed consent obtained from participants to store specimens and conduct future testing on diseases of public health importance to Nigeria.^12^ The NAIIS was a cross-sectional, two-stage, cluster survey designed to assess the prevalence of HIV and key health indicators; stage one consisted of probability proportional to size sampling of the enumeration area (EA) based on 2006 census household counts within each stratum (state and EA), and stage two consisted of randomly selected households within each selected EA. The overall size and distribution of the sample was determined by analysis of existing estimates of HIV incidence and prevalence in Nigeria.^12^ A random subset of households (equating to about every fourth household) were selected for pediatric sampling.^12^ A sample size of 32,354 children aged 0 to 14 years distributed across all 36 states and the Federal Capital Territory of Nigeria was estimated for the NAIIS survey. The specimens consisted of remnant plasma samples and DBS cards, which were stored in a central sample repository at Nigeria’s National Reference Laboratory.

### Multiplex bead assay.

The multiplex bead assay, including antigen coupling, DBS elution, and determination of cut points, has been described in detail elsewhere.^13^ In brief, DBS eluates were incubated at a 1:400 serum dilution with all bead-antigen sets for 1.5 hours. Soluble egg antigen was coupled to MagPlex microsphere beads (Luminex Corp., Austin, TX) in phosphate-buffered saline (PBS) at pH 7.2 using 120 μg protein for 12.5 × 106 beads.^11^ Total IgG was detected with 50 ng of biotinylated mouse anti-human IgG (Southern Biotech, Birmingham, AL), and IgG4 was detected with 40 ng of biotinylated mouse anti-human IgG4 (Southern Biotech) for 45 minutes, followed by incubation with 250 ng phycoerythrin-labeled streptavidin (Invitrogen, South San Francisco, CA) for 30 minutes. Supplemental anti-IgG4 was added (final concentration 200 ng/µL per well) because many chronic parasite infections such as schistosomiasis elicit strong IgG4 responses. Wells were incubated with PBS containing 0.5% bovine serum, albumin 0.02% sodium azide, and 0.05% Tween 20 for 30 minutes to remove any loosely bound antibodies. Beads were washed, resuspended in PBS, and read on a MAGPIX instrument equipped with xPONENT 4.1 software (Luminex Corp.). The median fluorescence intensity (MFI) with the background from the blank well subtracted out (MFI-BG) was recorded for each sample. The cut point for a positive SEA antibody result was considered an MFI-BG of 312 or greater and was based on the mean plus three SDs from a group of 86 adults from a non-endemic area. The sensitivity was 94% (95% CI: 82–98%), and the specificity was 97% (95% CI: 92–99%). Data were accepted only for wells in which 20 or more beads were read. *Schistosoma mansoni* SEA is similarly recognized by antibodies from persons with either *S. mansoni* and *S. haematobium* infections.^14^^,^^15^

### Questionnaire.

A questionnaire was administered to the head of the household that included demographic data for children residing in the household; water, sanitation, and hygiene (WASH) indicators; wealth indicators; animal ownership; and the child’s school attendance. A subset of these questions deemed relevant to schistosomiasis exposure risk were selected from the questionnaire for analysis. The WASH question responses were further categorized using established “improved” and “unimproved” type classifications^16^; improved sanitation systems are considered adequate for schistosomiasis control.^17^ Wealth scores, a measure of economic status, were determined based on data collected concerning dwelling and household characteristics, access to consumer goods and services, and household assets.^12^^,^^18^^,^^19^ Wealth scores were divided into quintiles, where quintile one represents the poorest and quintile five represents the wealthiest in the sample.

#### Statistical analyses.

Sampling weights were calculated by adjusting weights from the 2018 NAIIS based on state, 5-year age group, and sex to account for participants who did not consent or assent to store specimens, specimens that were unable to be assayed, or specimens that failed assay quality control. Statistical analyses accounted for the cluster survey design and sample stratification by state. SAS software version 9.4 (SAS Institute, Inc., Cary, NC) was used for all statistical analyses; only children who had been tested for antibodies to SEA were included in these analyses. *P *values less than 0.05 were considered significant for all analyses. Frequencies and proportions of risk factors were compared using the Rao-Scott χ^2^ test statistic.^20^ Univariate logistic regression using Taylor series linearization^21^ to account for cluster sampling was performed to calculate odds ratios and 95% CIs for variables significant on the χ^2^ test of association. Mean age of seropositive and seronegative children was compared using the least squares means statement in PROC SURVEYREG modeling age by seropositivity. Because of the likely influence of rural versus urban settings on the risk factors analyzed for schistosomiasis, selected variables were reexamined via domain analysis on logistic regression controlling for rural and urban residence; adjusted odds ratios and 95% CIs are reported.

### Ethics approvals.

For NAIIS, parents/guardians provided written informed consent for their children to participate in the survey and to store specimens for future studies. Written assent was also obtained from children aged 10 to 14 years. Both NAIIS and the additional testing using the stored specimens protocols were reviewed and approved by human subject reviewers at the National Health Research Ethics Committee of Nigeria, the University of Maryland Baltimore, and the U.S. Centers for Disease Control and Prevention (CDC) and was conducted consistent with applicable federal laws and CDC policy.†

## RESULTS

**Figure 1.**
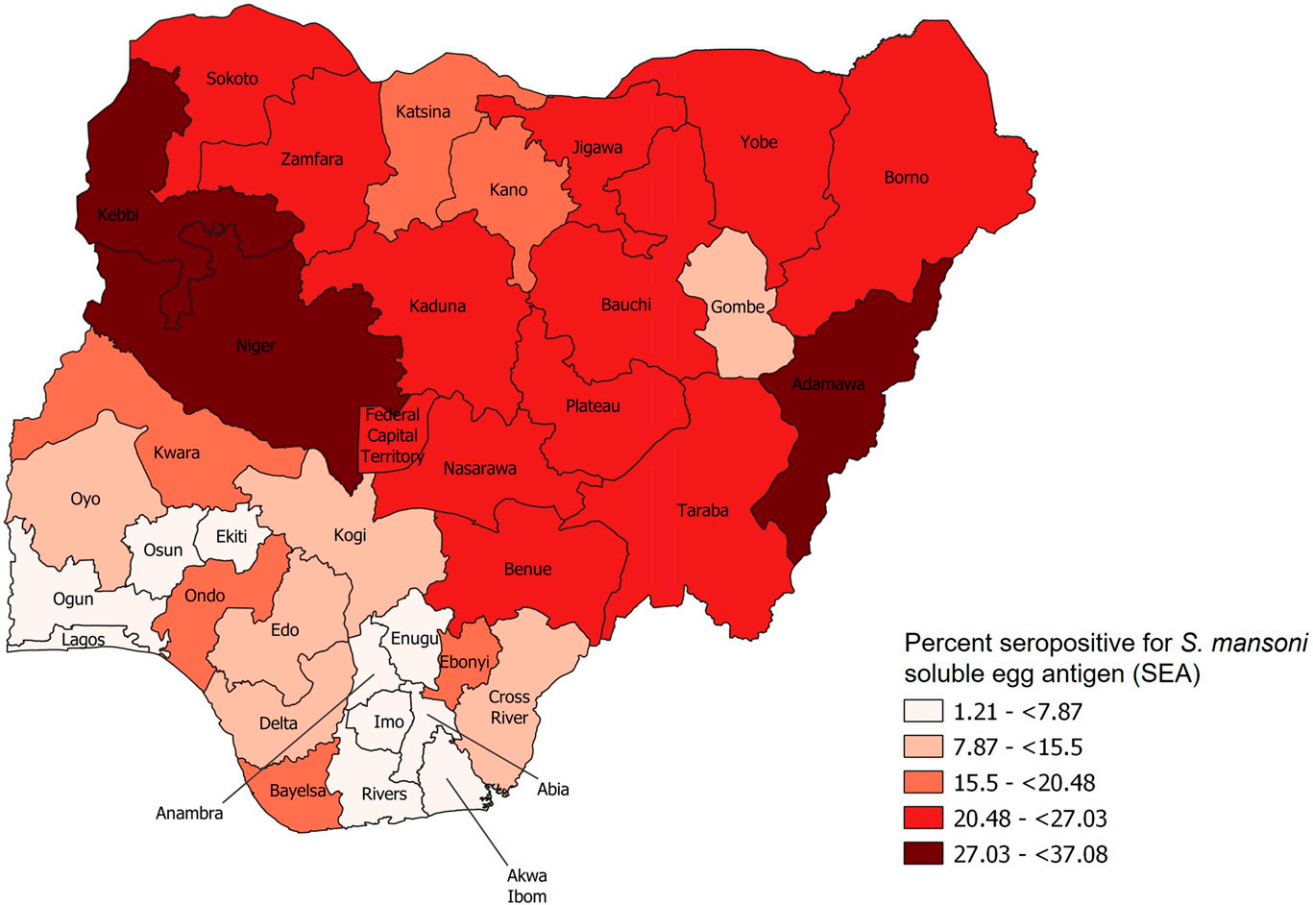
Seroprevalence by state in Nigeria, 2018.

A total of 32,494 children were enrolled in the study; SEA antibody data were available for 31,459 (96.8%). There were more boys (16,032; 51.3%) than girls (15,427; 48.7%) in the study sample, and more participants were from rural areas (18,449; 55.7%) than urban areas (13,010; 44.3%). The mean age was 7.3 years (range, 0–14 years). Overall seroprevalence was 17.2% (95% CI: 16.3–18.1%). By state, the prevalence of seropositive children ranged from 1.2% in Abia (95% CI: 0.3–2.1%) to 37.1% in Kebbi (95% CI: 30.8–43.3%). Figure 1 depicts weighted seroprevalence estimates by state for all 37 jurisdictions. Children from Kebbi had 48 times greater odds of seropositivity than children from Abia (crude odds ratio [cOR] = 48.1, 95% CI: 22.2–104.1) (Supplemental Table 1).

**Figure 2.**
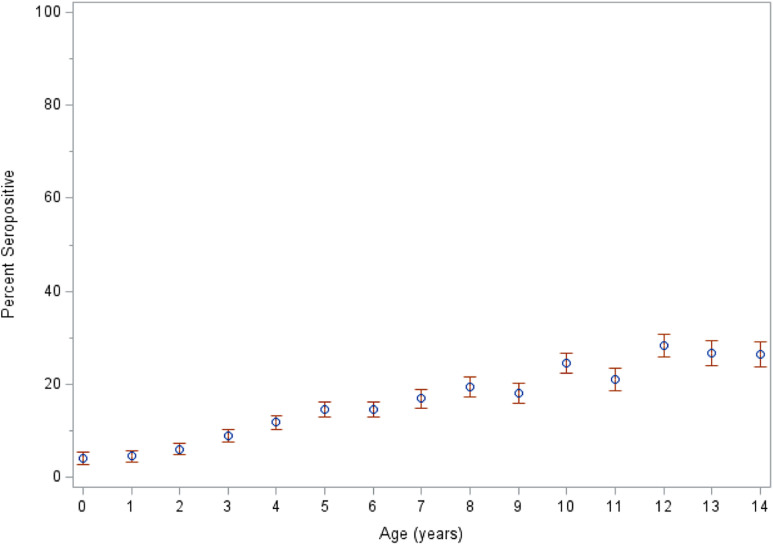
Weighted proportions of seropositive children in the sample by age (*N =* 31,459) in Nigeria, 2018. Error bars indicate 95% confidence intervals.

Seropositivity was significantly different between sexes, and boys had increased odds of seropositivity (cOR = 1.34, 95% CI: 1.24–1.45) (Table 1). Seropositive children also tended to be older: The mean age of seropositive children was 9.1 years compared with 7 years for seronegative children (*P* < 0.0001), and the weighted proportion of seropositive children in the sample increased with increasing age (Figure 2). There were seropositive children in every age group, including those less than 1 year of age (47/1,165; 4%). Seroprevalence was significantly different between urban and rural children, with rural children having increased odds of seropositivity (cOR = 2.2, 95% CI: 1.9–2.5) (Table 1). Children from households with access to improved sanitation facilities or improved drinking water sources had decreased odds of seropositivity (cOR = 0.52, 95% CI: 0.47–0.58 and cOR = 0.53, 95% CI: 0.47–0.60, respectively) (Table 1). Children from households with access to both improved sanitation and drinking water sources had the greatest reduction in odds of seropositivity (cOR = 0.38, 95% CI: 0.32–0.45).

**Table 1:** Demographics and risk factors of interest by serostatus among children aged 0 to 14 years in Nigeria, 2018

Variable	Seropositive (*n =* 5223)		Seronegative (*n =* 26,236)		*P*	Rao-Scott χ^2^ *F*-value	cOR (95% CI)
	*n*	% (CI) or mean (CI)	*n*	% (CI) or mean (CI)			
Sex							
Male (*n =* 16,032)	2,940	57.3 (55.5–59.1)	13,092	50.0 (49.3–50.7)	< 0.0001	52.03	1.34 (1.24–1.45)
Female (*n =* 15,427)	2,283	42.7 (40.9–44.5)	13,144	49.9 (49.2–50.7)	–	–	Ref
Age in years (*n =* 31,459)	5,223	9.1 (8.9–9.3)	26,236	7.0 (6.9–7.1)	< 0.0001	n/a	n/a
Urban (*n =* 13,010)	1,407	29.2 (25.7–32.7)	11,603	47.4 (44.9–49.8)	< 0.0001	138.41	Ref
Rural (*n =* 18,449)	3,816	70.8 (67.3–74.3)	14,633	52.6 (50.2–55.0)	–	–	2.2 (1.9–2.5)
Drinking water*							
Improved (*n =* 23,498)	3,327	64.6 (61.5–67.8)	20,171	77.6 (75.7–79.4)	<0.0001	50.86	0.53 (0.47–0.60)
Unimproved (*n =* 7,862)	1,876	34.9 (31.8–38.1)	5,986	22.1 (20.3–24.9)	–	–	Ref
Sanitation†							
Improved (*n =* 17,915)	2,292	44.7 (41.6–47.8)	15,623	60.7 (58.9–62.5)	<0.0001	78.95	0.52 (0.47–0.58)
Unimproved (*n =* 13,231)	2,880	54.4 (51.4–57.5)	10,351	38.4 (36.6–40.3)	–	–	Ref
Drinking water and sanitation							
Both improved (*n =* 15,402)	1,771	35.5 (32.6–38.3)	13,631	53.8 (51.9–55.8)	< 0.0001	76.79	0.38 (0.32–0.45)
Only one improved (*n =* 10,341)	2,034	39.0 (36.3–41.8)	8,307	31.4 (29.8–33.0)	–	–	0.72 (0.62–0.84)
Both unimproved (*n =* 5,319)	1,352	25.5 (22.5–28.5)	3,967	14.8 (13.2–16.3)	–	–	Ref
Wealth quintile							
1st (*n =* 6,676)	1,811	35.7 (32.6–38.7)	4,865	18.5 (17.0–20.1)	<0.0001	132.48	7.3 (6.0–8.8)
2nd (*n =* 6,523)	1,461	27.2 (24.8–29.5)	5,062	19.3 (17.9–20.6)	–	–	5.3 (4.4–6.5)
3rd (*n =* 6,556)	1,027	19.6 (17.6–21.5)	5,529	20.4 (19.2–21.6)	–	–	3.6 (3.0–4.4)
4th (*n =* 6,312)	638	12.2 (10.5–13.8)	5,674	21.2 (20.0–22.5)	–	–	2.2 (1.8–2.7)
5th (*n =* 5,392)	286	5.4 (4.5–6.4)	5,106	20.6 (19.3–21.9)	–	–	Ref
Child currently enrolled in school							
Yes (*n =* 20,536)	3,377	65.5 (63.2–67.9)	17,159	65.4 (64.3–66.6)	< 0.0001	53.11	Ref
No (*n =* 6,147)	1,422	26.9 (24.8–29.1)	4,725	18.1 (17.1–19.1)	–	–	1.48 (1.34–1.64)
No, too young (*n =* 4,711)	399	7.0 (6.1–7.9)	4,312	16.3 (15.5–17.0)	–	–	0.43 (0.37–0.50)
Child enrolled in school in previous year							
Yes (*n =* 16,591)	2,706	57.6 (55.2–60.0)	13,885	63.9 (62.5–65.3)	–	–	Ref
No (*n =* 9,860)	2,028	42.1 (39.7–44.5)	7,832	35.9 (34.5–37.3)	< 0.0001	11.14	1.30 (1.18–1.44)
Own animals or livestock							
Yes (*n =* 17,122)	3,316	64.3 (61.9–66.8)	13,806	51.8 (50.2–53.5)	< 0.0001	100.38	1.67 (1.52–1.85)
No (*n =* 14,309)	1,904	35.7 (33.2–38.2)	12,405	48.2 (46.5–49.8)	–	–	Ref
Which animals‡							
Cows/bulls (*n =* 3,276)	805	25.3 (22.1–28.5)	2,471	17.8 (16.0–19.6)	< 0.0001	13.1374	1.57 (1.35–1.82)
Other cattle (*n =* 3,059)	774	23.2 (20.2–26.3)	2,285	16.2 (14.5–17.9)	< 0.0001	21.4855	1.57 (1.34–1.83)
Horse/donkey/mule (*n =* 481)	130	3.7 (2.6–4.8)	351	2.6 (1.9–3.3)	0.0419	4.1436	1.45 (1.03–2.02)
Goats (*n =* 11,001)	2,317	69.8 (66.9–72.7)	8,684	62.4 (60.5–64.3)	< 0.0001	22.6042	1.39 (1.22–1.59)
Sheep (*n =* 6,087)	1,361	42.8 (39.5–46.1)	4,726	35.2 (33.3–37.1)	< 0.0001	18.8330	1.38 (1.21–1.56)
Poultry (*n =* 12,824)	2,512	74.5 (71.8–77.3)	10,312	73.4 (71.7–75.2)	0.0807	2.6056	1.03 (0.89–1.18)
Pigs (*n =* 814)	215	5.6 (3.9–7.4)	599	3.9 (3.1–4.7)	0.0563	3.6476	1.46 (1.03–2.06)
Camels (*n =* 223)	68	1.9 (1.2–2.8)	155	1.2 (0.8–1.6)	0.0354	4.4300	1.69 (1.11–2.56)
Dogs (*n =* 2,497)	534	15.3 (12.9–17.8)	1,963	13.4 (12.2–14.7)	0.1034	2.6550	1.17 (0.98–1.39)

cOR = crude odds ratio; n/a = not applicable; Ref = reference category; UNICEF = United Nations Children’s Fund.

* Drinking water sources were categorized as “improved” and “unimproved” according to WHO and UNICEF guidelines. Improved drinking water sources include piped into dwelling, piped to yard/plot, public tap/standpipe, piped to neighbor, tube well or borehole, protected well, protected spring, tanker truck, cart with small tank/jerry can/cartless vendor, bottled water/dispenser water, sachet (pure) water, rainwater. Unimproved sources include unprotected wells and springs and surface water.

† Sanitation responses were categorized as “improved” and “unimproved” according to WHO and UNICEF guidelines. Improved sanitation systems include flush to piped sewer system, flush to septic tank, flush to pit latrine, flush to don’t know where, pit latrine with slab, and composting toilet. Unimproved systems include pit latrine without slab/open pit, bucket toilet, hanging toilet/hanging latrine, no facility/bush/field, flush to somewhere else, and ventilated improved pit latrine.

‡ Sample sizes listed are the total number of respondents who reported owning at least one of the animal type listed.

Animal/livestock ownership was significantly associated with seropositivity, and children from households who reported owning livestock had increased odds of schistosomiasis seropositivity compared with children from households who did not (cOR = 1.67, 95% CI: 1.52–1.85). Independently, children from households reporting ownership of cows or bulls, other cattle, goats, sheep, or camels had greater odds of seropositivity than children from households who did not own these types of livestock (Table 1).

The effect of school attendance during the year when the sampling was conducted and the year prior to sampling was also assessed (Table 1). Children who were not currently enrolled in school or had not been enrolled in the previous year had greater odds of seropositivity (cOR = 1.48, 95% CI: 1.34–1.64 and cOR = 1.30, 95% CI: 1.18–1.44, respectively). Wealth scores, divided into quintiles, were significantly associated with seropositivity (Rao Scott χ^2^ *F*-value = 132.48, *P <* 0.0001), with a smaller proportion of seropositive children from families with higher wealth scores (Table 1). Children from households in each quintile below the highest wealth scores (quintile 5) had increasing odds of seropositivity; children from households with the lowest wealth scores (quintile 1) had 7.3 times greater odds of seropositivity (95% CI: 6.04–8.81) than children from households with the highest wealth scores (Table 1).

Because of the likely influence of rural or urban settings on the risk factors analyzed for schistosomiasis, all variables were reexamined via domain analysis separating rural and urban into two subpopulations. When geographical location by state was examined, the odds of seropositivity differed greatly between rural and urban children, with rural children generally having increased odds of seropositivity compared with urban children (Supplemental Table 2). Notably, in the states of Enugu, Ekiti, Ebonyi, Cross River, Akwa Ibom, Rivers, Delta, Edo, Ogun, and Lagos, the odds of seropositivity were significantly elevated for rural children, but were nonsignificant for urban children (Supplemental Table 2). More children from urban households had access to improved sanitation facilities (*n =* 10,119; 77.8%) and improved drinking water sources (*n =* 12,019; 92.3%) than children from rural households (Rao Scott χ^2^ *F*-value = 252.54, *P <* 0.0001; Rao Scott χ^2^ *F*-value = 188.9, *P <* 0.001, respectively) (Table 2). Access to both improved sanitation facilities and drinking water sources were associated with decreased odds of seropositivity regardless of whether children lived in rural or urban areas (Table 2). Likewise, animal/livestock ownership also differed significantly between urban and rural households, with a greater proportion of rural households reporting animal ownership (66% compared with 38.7%, Rao Scott χ^2^ *F*-value = 207.1, *P* < 0.0001), but the odds of seropositivity remained increased among children whose households reported animal ownership regardless of whether they lived in rural or urban areas (Table 2).

**Table 2:** Effect of rural versus urban location of home on seropositivity in relation to selected risk factors in Nigeria, 2018

Variable	Urban (*n =* 13,010)		Rural (*n =* 18,449)		*P*	Rao-Scott χ^2^ *F*-value	aOR (95% CI) urban	aOR (95% CI) rural
	*n*	% (CI) or mean (CI)	*n*	% (CI) or mean (CI)				
Seropositive	1,407	11.4 (10.2–12.5)	3,816	21.9 (20.6–23.2)	< 0.0001	138.41	n/a	n/a
Seronegative	11,603	88.6 (87.5–89.8)	14,633	78.1 (76.8–79.4)	–	–	–	–
Drinking water								
Improved (*n =* 23,498)	12,019	92.3 (90.7–93.9)	11,479	61.9 (59.1–64.6)	< 0.0001	188.95	0.5 (0.4–0.6)	0.7 (0.6–0.8)
Unimproved (*n =* 7,862)	952	7.4 (5.9–9.0)	6,910	37.8 (35.0–40.5)	–	–	Ref	Ref
Sanitation								
Improved (*n =* 17,915)	10,119	77.8 (75.7–79.9)	7,796	42.2 (39.7–44.7)	< 0.0001	252.54	0.6 (0.5–0.7)	0.7 (0.6–0.8)
Unimproved (*n =* 13,231)	2,851	21.8 (19.7–23.9)	10,380	56.6 (54.1–59.1)	–	–	Ref	Ref
Child currently enrolled in school								
Yes (*n =* 20,536)	9,764	74.7 (73.2–76.2)	10,772	58.1 (56.5–59.8)	< 0.0001	61.94	Ref	Ref
No (*n =* 6,147)	1,511	12.4 (11.0–13.8)	4,636	25.4 (23.8–27.0)	–	–	1.1 (0.9–1.5)	1.3 (1.2–1.5)
No, too young (*n =* 4,711)	1,727	12.8 (12.0–13.6)	2,984	16.2 (15.2–17.1)	–	–	0.4 (0.3–0.5)	0.4 (0.3–0.5)
Child enrolled in school in previous year								
Yes (*n =* 16,591)	8,178	72.4 (70.5–74.3)	8,413	54.7 (52.8–56.7)	< 0.0001	93.84	Ref	Ref
No (*n =* 9,860)	3,020	27.5 (25.6–29.4)	6,840	45.1 (43.1–47.0)	–	–	1.1 (0.9–1.3)	1.1 (1.0–1.3)
Own animals or livestock								
Yes (*n =* 17,122)	5,110	38.7 (36.2–41.1)	12,012	66.1 (64.1–68.1)	< 0.0001	207.08	1.6 (1.4–1.9)	1.3 (1.2–1.5)
No (*n =* 14,309)	7,890	61.2 (58.8–63.7)	6,419	33.8 (31.9–35.8)	–	–	Ref	Ref
Wealth quintile								
1st (*n =* 6,676)	907	7.2 (5.4–9.0)	5,769	32.8 (30.5–35.2)	< 0.0001	274.06	6.8 (4.7–9.8)	5.2 (3.5–7.8)
2nd (*n =* 6,523)	1,221	10.3 (8.8–11.8)	5,302	28.8 (26.9–30.7)	–	–	5.0 (3.8–6.6)	3.9 (2.6–5.8)
3rd (*n =* 6,556)	2,475	19.0 (17.2–20.7)	4,081	21.3 (19.6–22.9)	–	–	3.4 (2.7–4.4)	2.8 (1.8–4.2)
4th (*n =* 6,312)	3,892	28.7 (26.7–30.6)	2,420	12.5 (11.3–13.7)	–	–	2.2 (1.7–2.8)	1.7 (1.1–2.5)
5th (*n =* 5,392)	4,515	34.9 (32.6–37.2)	877	4.6 (3.8–5.3)	–	–	Ref	Ref

aOR = adjusted odds ratio; n/a = not applicable; Ref = reference category.

## DISCUSSION

The overall national weighted schistosomiasis seroprevalence among children aged 0 to 14 years in Nigeria determined in this study was 17.2%. To our knowledge, this is the first nationally representative estimate of seroprevalence based on direct sampling. This estimate differs from other recently published national estimates of schistosomiasis prevalence in Nigeria, which range from 27.9%^3^ to 32.1%.^22^ Differences in methodology and study population are likely responsible for some of the discrepancy. Ekpo et al.^3^ compiled national- and state-level prevalence estimates utilizing data gathered from literature review, the WHO, and the United Nations Children’s Fund (UNICEF), as well as survey data obtained by Nigerian government entities, nongovernmental organizations, universities, and other local research institutions going back as far as 1960; their estimates encompass all schistosome species. Odeniran et al.^22^ also performed a meta-analysis of published studies between 1983 and 2018 but focused only on the prevalence of *S. haematobium* infections. Both studies’ prevalence estimates reflect study populations of children aged 5 to 14 years. Published studies, such as those incorporated into the meta-analyses of both Ekpo et al. and Odeniran et al. are largely directed research efforts targeting specific areas of increased disease risk; analyses using these data points may yield higher prevalence estimates. Schistosomiasis is a focal disease, dependent upon environmental factors suitable to maintain snail intermediate-host populations, contamination of water sources by human waste, and human water contact behavior. For example, in western Kenya, there is a clear relationship between schistosomiasis prevalence and distance to Lake Victoria,^23^ which is the primary source of infection in that area. In one study examining schistosomiasis seroprevalence rates among children from communities near Lake Victoria, over 90% of seropositive children lived within 1.5 km of the lake.^9^ Because the present seroprevalence study was designed to be representative of the Nigerian population and to determine HIV incidence and not to capture the heterogeneity in schistosomiasis across the country or detect hotspots, there may have been highly focal areas of schistosomiasis infection that were not included in the study population and resulted in a lower prevalence estimate than those identified in the meta-analyses. However, the study design used here is considered the most robust for producing a national prevalence estimate, and the 95% CI for the resulting national-level seroprevalence estimate is narrow (16.3–18.1%), indicating that the sample size should be adequate. The previously published meta-analyses also considered studies of schistosomiasis in Nigeria published over many years. As our study focused on seroprevalence among children aged 0 to 14 years, it reflects more recent transmission; thus, another potential explanation in the differing prevalence estimates is that Nigeria’s efforts to control schistosomiasis may have decreased infection rates in some areas.

In this study, boys, increasing age, residence in a rural area, lack of improved sanitation and drinking water sources, animal ownership, and low wealth scores were all associated with increased odds of seropositivity. Boys, age, lack of potable water, and low wealth scores are consistent with risk factors identified in previous studies.^22^^,^^24^^,^^25^ There were seropositive children identified in every age group, including among infants and preschool–aged children (2–5 years), similar to findings from other studies both in Nigeria^26^ and from other schistosomiasis-endemic countries in Africa.^11^^,^^27^ Maternal IgG SEA antibody wanes by 20 weeks of age; thus, any increase in neonatal antibody beyond this point would be indicative of acquired infection.^27^ Infants may be exposed to schistosome-contaminated waters through bathing, whereas preschool-aged children may be exposed during bathing, swimming, or when accompanying older children and adults to water sources for domestic chores.^26^ Schistosomiasis seroprevalence increased with age, likely because of accumulating exposures over time. School enrollment was associated with decreased odds of seropositivity. This may be because children who are in school would likely have received education or treatment through school-based deworming campaigns, which is Nigeria’s main method of control for schistosomiasis,^28^ or because being in school may preclude their participating in activities (e.g., for recreation, work, or domestic chores) likely to put them at risk of schistosomiasis.

Regardless of whether the child lived in a rural or urban area, schistosomiasis seropositivity was associated with unimproved toilet facilities and unimproved drinking water sources. Unimproved toilet types contribute to ongoing transmission of schistosomiasis as they allow human urine and feces to contaminate water sources where the life cycle will continue if appropriate snail intermediate hosts are present. Collection of drinking water from an unimproved source that could be contaminated with schistosome cercaria is likely a more important contributor to disease transmission, rather than consumption of the contaminated water itself.^17^ Improvements in water supplies and sanitation that limit human contact with contaminated water, for example, by providing not only safe drinking water but also laundry and bathing facilities, greatly reduce infection rates, especially when combined with preventive chemotherapy.^17^^,^^29^ Studies similar to this one may help provide information on what level of improved water and sanitation are needed to observe an impact on schistosomiasis prevalence.

Interestingly, animal ownership was associated with increased odds of seropositivity, and this did not differ between urban or rural households. Although some schistosomes are zoonotic and bovid schistosomes are capable of hybridizing with *S. haematobium* and infecting humans in sub-Saharan Africa,^30^^–^^33^ the transmission of disease from animals to humans is not direct. Schistosomes have a two-host life cycle with an intermediate snail host; eggs shed in stool or urine are not immediately infective and must undergo development within the snail intermediate host to become infective cercariae. Instead, the association of animal ownership with seropositivity may be attributable to increased contact with potentially contaminated water sources owing to the need to either collect water for the animals or driving animals to water sources. It is unknown whether the antigen used in this analysis (*S. mansoni* SEA) would detect antibody to hybridized *S. haematobium–Schistosoma bovis* infection, although sera from persons with *S. haematobium* infections do react with *S. mansoni* SEA. To our knowledge, this is the first time human schistosomiasis prevalence measures have been studied in relation to animal or livestock ownership.

Serological studies for neglected tropical diseases such as schistosomiasis can contribute important information but also have limitations. The population-based survey methodology used here was not designed to identify focal areas of schistosomiasis transmission. No stool or urine was collected, and no other testing for schistosomiasis was done that could be used to correlate or compare prevalence estimates. Antibodies identified in seroprevalence studies only measure exposure to a pathogen and do not necessarily indicate active disease. Thus, seroprevalence studies cannot discern between active and former infections, as individuals may still retain antibodies for many years after their infection has cleared.^34^ The SEA made from *S. mansoni* eggs does not distinguish between infections caused by *S. haematobium* and *S. mansoni*.^14^^,^^15^ In addition, *S. intercalatum* sera tested by ELISA in our laboratories demonstrated reactivity to *S. mansoni* SEA. We expect the multiplex bead assay would also detect reactivity in persons with *S. intercalatum* infections, but this has not yet been directly tested. It should also be noted that the state of residence reported during the NAIIS survey may not reflect the actual location of exposure, as neither migration nor travel was measured during the survey. Although not a limitation of seroprevalence studies for schistosomiasis, a disadvantage is that the opportunity to also detect or monitor soil-transmitted helminth infections is lost if stools are not collected.

The practice of “piggybacking” on a large population-based study conducted for another purpose may not capture the full breadth of risk factors important to other diseases. The questionnaire used during the NAIIS did not capture other exposures to water such as recreational (e.g., swimming), occupational (e.g., fishing, car washing), or for other purposes (e.g., washing, laundry) that would put a person at risk of schistosome infection. But it may introduce the opportunity to identify new risk factors not previously evaluated in targeted studies, such as the animal/livestock ownership risk factor observed here. Nevertheless, longitudinal monitoring of seroprevalence could provide information on changes in exposure or potentially the success (or failure) of prevention and control efforts. For example, monitoring seroprevalence levels among infants and preschool-aged children could more directly measure the force of transmission in an area, as infections in these age groups reflect recent transmission and may also provide relative prevalence in older age groups.^9^ Population-based seroprevalence studies such as these can eventually offer information to support the achievement of interruption of transmission in endemic areas.^35^

Children infected with schistosomes can suffer from stunted growth, chronic anemia, decreased aerobic capacity, and cognitive deficits, as well as the direct effects of chronic schistosomiasis infection: hepatic fibrosis, obstructive uropathy, and complications of urogenital infections, which may include an increased risk of female genital schistosomiasis, HIV, squamous cell carcinoma, and cervical cancer.^4^^,^^36^ The results of this seroprevalence study can provide valuable information for controlling schistosomiasis in Nigeria, particularly with respect to age and geographic distributions. Seropositive children were detected in every age group, including among infants and preschool-aged children not targeted by Nigeria’s school-based prevention and control programs.^28^ The new WHO guidelines, released in February 2022, now call for annual preventive chemotherapy in all age groups, including children aged 2 years or older, in communities where the prevalence is at least 10% determined by parasitological methods, which would likely include many states in Nigeria.^37^ These new guidelines also recommend treatment of infected infants less than 2 years of age in health facilities, regardless of the community prevalence level.^37^ The differences in seropositivity between urban and rural children highlight that although urban children are still being exposed to schistosomiasis, rural children have significantly increased odds of seropositivity, meaning their exposure is likely much greater than that of urban children. This was true in almost all states in Nigeria and could provide an indication of where to direct resources. Improved drinking water and sanitation facilities were associated with decreased odds of seropositivity regardless of whether the child resided in a rural or urban area, highlighting the importance of these measures in preventing schistosomiasis exposure. The WHO’s new guidelines also recommend the implementation of WASH interventions in endemic areas, calling them “essential measures” to reduce transmission.^37^ Animal ownership, although more common among rural families, was also associated with increased odds of seropositivity regardless of whether the child lived in a rural or urban area. This may be an important finding to consider when provisioning WASH interventions and water improvement projects, as it may be necessary to also provide safe water sources for animals to prevent exposure among livestock herders/owners and further decrease transmission. Health education about schistosomiasis transmission and water contact should also be targeted to those who own livestock or tend to them.

## Supplemental files

10.4269/ajtmh.23-0219 Supplemental Materials

## Footnotes

† 45 C.F.R. part 46; 21 C.F.R. part 56.
