# Supplementary material for: Schistosomiasis Seroprevalence among Children Aged 0–14 Years in Nigeria, 2018
Source: Am J Trop Med Hyg. 2023 Nov 27;110(1):90–7. doi: 10.4269/ajtmh.23-0219 (PMC10793028; doi:10.4269/ajtmh.23-0219)
Supplement: Supplemental Materials [file tpmd230219.SD1.pdf]

Supplementary Table 1: Comparative seroprevalence estimates by states in Nigeria, 2018.

Variable	Seropositive (n=5223)		Seronegative (n=26236)		p-value	Rao-Scott χ^2 F-value	cOR (95% CI)
	n	% (CI) or mean (CI)	n	% (CI) or mean (CI)			
State					<0.0001	13.8835	
Abia (n=646)	8	1.2 (0.3 – 2.1)	638	98.8 (97.9 – 99.7)			Ref
Enugu (n=679)	15	2.2 (0.9 – 3.5)	664	97.8 (96.5 – 99.1)			1.8 (0.7 – 4.7)
Imo (n=1092)	24	2.3 (1.0 – 3.5)	1068	97.7 (96.5 – 98.9)			1.9 (0.8 – 4.7)
Ekiti (n=326)	9	2.6 (0.4 – 4.9)	317	97.4 (95.1 – 99.6)			2.2 (0.7 – 6.9)
Anambra (n=884)	23	2.8 (1.3 – 4.4)	861	97.2 (95.6 – 98.7)			2.4 (0.9 – 6.0)
Ogun (n=603)	37	5.5 (3.1 – 7.9)	566	94.5 (92.1 – 96.9)			4.8 (2.0 – 11.3)
Lagos (n=1671)	116	5.7 (4.4 – 7.1)	1555	94.3 (92.9 – 95.6)			4.9 (2.3 – 10.7)
Akwa Ibom (n=794)	53	6.1 (4.3 – 7.9)	741	93.9 (92.1 – 95.7)			5.3 (2.4 – 11.6)
Osun (n=443)	32	7.2 (4.9 – 9.5)	411	92.8 (90.5 – 95.1)			6.3 (2.8 – 14.2)
Rivers (n=941)	63	7.9 (4.6 – 11.1)	878	92.1 (88.9 – 95.3)			7.0 (3.0 – 16.3)
Edo (n=660)	84	11.7 (5.4 – 17.9)	576	88.3 (82.0 – 94.6)			10.8 (4.2 – 27.8)
Delta (n=916)	107	12.1 (9.0 – 15.1)	809	87.9 (84.9 – 90.9)			11.2 (5.1 – 24.4)
Gombe (n=826)	102	13.3 (9.2 – 17.4)	724	86.7 (82.6 – 90.8)			12.5 (5.6 – 28.0)
Kogi (n=506)	73	14.2 (8.2 – 20.2)	433	85.8 (79.8 – 91.8)			13.5 (5.6 – 32.5)
Oyo (n=1024)	133	14.9 (7.5 – 22.3)	891	85.1 (77.7 – 92.5)			14.3 (5.6 – 36.2)
Cross River (n=605)	78	15.5 (10.0 – 20.9)	527	84.5 (79.0 – 89.9)			15.0 (6.5 – 34.5)
Kano (n=1835)	308	18.2 (13.7 – 22.7)	1527	81.8 (77.3 – 86.3)			18.1 (8.3 – 39.7)
Kwara (n=426)	71	18.2 (12.1 – 24.2)	355	81.8 (75.8 – 87.9)			18.1 (7.9 – 41.6)
Ebonyi (n=700)	115	18.2 (12.5 – 23.9)	585	81.8 (76.0 – 87.5)			18.2 (8.0 – 41.3)
Katsina (n=1621)	284	18.4 (14.9 – 21.9)	1337	81.6 (78.1 – 85.0)			18.4 (8.6 – 39.4)
Bayelsa (n=471)	84	19.3 (12.6 – 25.9)	387	80.7 (74.1 – 87.4)			19.4 (8.4 – 45.1)
Ondo (n=541)	93	20.5 (14.6 – 26.3)	448	79.5 (73.7 – 85.4)			21.0 (9.3 – 47.1)
Nasarawa (n=430)	87	22.2 (15.0 – 29.3)	343	77.8 (70.7 – 84.9)			23.2 (10.1 – 53.5)
FCT (n=237)	47	22.5 (14.3 – 30.6)	190	77.5 (69.4 – 85.7)			23.6 (10.0 – 55.9)
Borno (n=641)	154	22.5 (17.6 – 27.4)	487	77.5 (72.6 – 82.4)			23.6 (10.9 – 51.4)
Benue (n=939)	216	22.8 (18.6 – 26.9)	723	77.2 (73.1 – 81.4)			24.0 (11.2 – 51.5)
Kaduna (n=1836)	404	23.1 (17.8 – 28.4)	1432	76.9 (71.6 – 82.2)			24.5 (11.2 – 53.7)
Taraba (n=694)	152	23.5 (19.0 – 27.9)	542	76.5 (72.1 – 81.0)			25.0 (11.6 – 53.8)
Jigawa (n=1386)	313	24.2 (20.0 – 28.4)	1073	75.8 (71.6 – 80.0)			26.0 (12.2 – 55.7)
Plateau (n=784)	185	24.8 (20.0 – 29.6)	599	75.2 (70.4 – 80.0)			26.9 (12.5 – 58.1)
Yobe (n=583)	147	25.2 (20.0 – 30.4)	436	74.8 (69.6 – 80.0)			27.5 (12.7 – 59.7)
Sokoto (n=857)	212	25.2 (20.0 – 30.4)	645	74.8 (69.6 – 79.9)			27.5 (12.7 – 59.7)
Zamfara (n=375)	100	25.8 (18.9 – 32.6)	275	74.2 (67.4 – 81.0)			28.3 (12.6 – 63.5)
Bauchi (n=1435)	370	27.0 (22.1 – 31.9)	1065	72.9 (68.1 – 77.9)			30.2 (14.0 – 65.0)
Niger (n=1312)	374	29.7 (24.3 – 35.0)	938	70.4 (65.0 – 75.7)			34.4 (15.9 – 74.1)
Adamawa (n=866)	242	31.1 (24.3 – 37.9)	624	68.9 (62.1 – 75.7)			36.9 (16.7 – 81.3)
Kebbi (n=874)	308	37.1 (30.8 – 43.3)	566	62.9 (56.7 – 69.2)			48.1 (22.2 – 104.1)

Horizontal line delineates national seroprevalence estimate of 17.2% determined in this study. FCT=Federal Capital Territory

Supplementary Table 2: Effect of rural vs. urban location of the home on seroprevalence estimates by state in Nigeria, 2018.

Variable	Urban (n=13010)		Rural (n=18449)		p-value	Rao-Scott χ^2 F-value	aOR (95% CI) Urban	aOR (95% CI) Rural
	n	% (CI)	n	% (CI)				
State								
Abia (n=646)	197	29.0 (17.8 – 40.1)	449	71.0 (59.9 – 82.2)	<0.0001	17.66	Ref	Ref
Enugu (n=679)	171	21.9 (11.8 – 32.1)	508	78.1 (67.9 – 88.2)			0.5 (0.1 – 3.0)*	3.5 (1.1 – 11.3)
Imo (n=1092)	341	31.0 (19.5 – 42.4)	751	69.0 (57.6 – 80.5)			2.1 (0.6 – 7.2)	1.5 (0.4 – 5.4)
Ekiti (n=326)	254	79.6 (68.6 – 90.6)	72	20.4 (9.4 – 31.4)			0.8 (0.2 – 2.9)*	8.3 (1.2 – 58.8)
Anambra (n=884)	720	83.3 (73.2 – 93.4)	164	16.7 (6.6 – 26.8)			1.2 (0.4 – 3.9)	4.0 (0.9 – 17.4)
Ogun (n=603)	411	65.8 (54.3 – 77.4)	192	34.2 (22.6 – 45.7)			1.5 (0.5 – 4.6)*	14.3 (4.2 – 48.0)
Lagos (n=1671)	1454	93.2 (90.6 – 95.8)	217	6.8 (4.2 – 9.4)			2.1 (0.7 – 5.9)*	27.6 (9.2 – 82.4)
Akwa Ibom (n=794)	96	14.7 (6.1 – 23.4)	698	85.3 (76.6 – 93.9)			1.8 (0.5 – 7.5)*	9.3 (3.3 – 26.5)
Osun (n=443)	396	87.5 (78.8 – 96.2)	47	12.5 (3.8 – 21.2)			3.2 (1.1 – 9.1)	11.1 (2.7 – 45.1)
Rivers (n=941)	292	30.7 (20.0 – 41.3)	649	69.3 (58.7 – 80.0)			2.4 (0.5 – 11.1)*	13.4 (4.5 – 40.0)
Edo (n=660)	348	56.3 (43.8 – 68.8)	312	43.7 (31.2 – 56.2)			2.5 (0.8 – 7.6)*	32.9 (9.2 – 117.9)
Delta (n=916)	393	39.0 (27.1 – 50.8)	523	61.0 (49.2 – 72.9)			2.5 (0.8 – 7.4)*	26.4 (9.4 – 73.9)
Gombe (n=826)	271	32.0 (18.1 – 45.9)	555	68.0 (54.1 – 81.9)			3.9 (1.3 – 11.8)	24.9 (8.4 – 73.4)
Kogi (n=506)	297	55.3 (41.0 – 69.5)	209	44.7 (30.5 – 59.0)			8.5 (2.6 – 28.6)	15.9 (5.3 – 47.3)
Oyo (n=1024)	716	67.6 (55.7 – 79.4)	308	32.4 (20.6 – 44.3)			3.8 (1.3 – 10.9)	53.7 (13.4 – 214.2)
Cross River (n=605)	88	12.2 (4.2 – 20.1)	517	87.8 (79.9 – 95.8)			0.8 (0.2 – 4.4)*	28.7 (9.8 – 84.1)
Kano (n=1835)	1190	61.0 (44.5 – 77.5)	645	39.0 (22.5 – 55.5)			7.0 (2.3 – 20.8)	42.5 (14.8 – 121.9)
Kwara (n=426)	188	45.2 (29.6 – 60.7)	238	54.8 (39.3 – 70.4)			5.0 (1.6 – 15.6)	43.6 (14.7 – 129.8)
Ebonyi (n=700)	120	17.9 (8.4 – 27.5)	580	82.1 (72.5 – 91.6)			3.0 (0.8 – 11.7)*	35.7 (12.3 – 103.9)
Katsina (n=1621)	226	14.8 (5.4 – 24.2)	1395	85.2 (75.8 – 94.6)			4.0 (1.3 – 12.0)	34.3 (12.4 – 95.0)
Bayelsa (n=471)	96	20.2 (10.1 – 30.3)	375	79.8 (69.7 – 89.9)			3.9 (1.1 – 14.0)	38.3 (12.9 – 114.4)
Ondo (n=541)	297	47.8 (34.8 – 60.8)	244	52.2 (39.2 – 65.2)			4.6 (1.6 – 13.7)	58.2 (19.8 – 170.8)
Nasarawa (n=430)	115	26.9 (13.4 – 40.4)	315	73.1 (59.6 – 86.6)			13.2 (3.5 – 49.6)	37.1 (12.5 – 110.4)
FCT (n=237)	224	93.7 (88.4 – 99.1)	13	6.3 (0.9 – 11.6)			11.1 (3.7 – 34.0)	79.7 (11.9 – 535.5)
Borno (n=641)	441	70.7 (54.0 – 87.3)	200	29.3 (12.7 – 46.0)			11.4 (3.9 – 33.1)	43.1 (15.2 – 122.5)
Benue (n=939)	158	12.3 (4.9 – 19.7)	781	87.7 (80.3 – 95.1)			8.9 (2.7 – 29.3)	41.8 (15.1 – 116.1)
Kaduna (n=1836)	1062	59.2 (46.4 – 71.9)	774	40.8 (28.1 – 53.6)			8.9 (3.1 – 25.4)	60.3 (20.0 – 181.9)
Taraba (n=694)	114	15.1 (5.0 – 25.1)	580	84.9 (74.9 – 95.0)			8.1 (2.7 – 24.7)	44.8 (16.1 – 124.8)
Jigawa (n=1386)	722	53.3 (40.7 – 66.0)	664	46.7 (34.0 – 59.3)			10.2 (3.6 – 29.4)	55.7 (19.9 – 155.9)
Plateau (n=784)	277	30.9 (19.5 – 42.3)	507	69.1 (57.7 – 80.5)			8.9 (3.1 – 25.2)	52.9 (18.7 – 150.1)
Yobe (n=583)	144	28.9 (13.0 – 44.8)	439	71.1 (55.2 – 87.0)			6.6 (1.8 – 24.2)	58.1 (21.1 – 160.0)
Sokoto (n=857)	236	30.3 (16.6 – 43.9)	621	69.7 (56.1 – 83.4)			9.4 (3.1 – 28.6)	53.3 (18.8 – 151.1)
Zamfara (n=375)	145	39.5 (22.3 – 56.7)	230	60.5 (43.3 – 77.7)			12.6 (4.1 – 38.6)	51.2 (17.0 – 154.3)
Bauchi (n=1435)	144	11.6 (4.2 – 19.0)	1291	88.4 (81.0 – 95.8)			6.9 (2.1 – 22.5)	54.9 (19.8 – 152.7)
Niger (n=1312)	224	13.0 (5.7 – 20.3)	1088	87.0 (79.7 – 94.3)			9.7 (2.7 – 34.9)	62.0 (22.2 – 173.0)
Adamawa (n=866)	254	25.0 (14.0 – 35.9)	612	75.0 (64.1 – 86.0)			9.7 (3.0 – 31.3)	73.9 (25.8 – 211.5)
Kebbi (n=874)	188	21.4 (10.1 – 32.7)	686	78.6 (67.3 – 89.9)			10.4 (3.3 – 32.9)	97.5 (34.9 – 272.6)

*Indicates states where the odds of seropositivity were non-significant for urban children but significantly increased for rural children. FCT=Federal Capital Territory
